# A core competency framework for mental health peer supporters of older adults in a Chinese community: cross-culturally informed Delphi study

**DOI:** 10.1192/bjb.2023.45

**Published:** 2024-04

**Authors:** Edwin Lok Yan Wong, Jessie Ho-Yin Yau, Lesley Cai Yin Sze, Hotinpo Sky Kanagawa, Dara Kiu Yi Leung, Tianyin Liu, Gloria Hoi Yan Wong, Terry Yat Sang Lum

**Affiliations:** 1 Department of Social Work and Social Administration, The University of Hong Kong, Hong Kong; 2 Sau Po Centre on Ageing, The University of Hong Kong, Hong Kong

**Keywords:** Psychological well-being, peer support, Delphi method, service users, core competencies

## Abstract

**Aims and method:**

Non-Western literature on the core competencies of mental health peer supporters remains limited. Therefore, we used a three-round Delphi study with peer supporters, service users (i.e. someone using peer support services) and mental health professionals to develop a core competency framework for peer supporters in the Chinese context.

**Results:**

The final framework included 35 core competencies, the conceptual origins of which were local (14.3%), Western (20%) and both local and Western (65.7%). They were grouped into five categories in ascending peer supporter role specificity: (1) self-care and self-development, (2) general work ethics, (3) work with others, (4) work with service users and (5) peer support knowledge.

**Clinical implications:**

A culturally valid mental health peer support competency framework can minimise role confusion and refine training and practice guidelines. In a Chinese context, peer supporters were valued as generic support companions, whereas functions highlighted in the West, such as role modelling, were perceived as less critical.

Peer support is widely defined as social and emotional support offered by individuals to others sharing a similar background or health condition that engenders a desired personal change.^1,2^ Recent years have witnessed population ageing worldwide, generating increased demands for peer support programmes to improve the mental well-being of older adults and bridge the divide between helping professionals and service users in mental health interventions^3–5^ and for professional or standardised development of peer support to ensure higher service quality and ease of practice.^6,7^ As peer supporters work in diverse settings and embrace multiple service roles, a long-standing challenge has been role confusion or conflict generating uncertainty regarding their core competencies.^7–9^ For peer supporters, ‘competencies’ are the bundles of necessary qualities, knowledge, skills and attitudes that an individual should possess to fulfil peer support roles,^10,11^ and associated competency frameworks have been developed.^11,12^ The Delphi method has been used to examine stakeholder consensus on critical aspects of peer support in particular service contexts.^13–15^

Limited literature has investigated peer supporter competencies considered important for the mental healthcare of older adults or the perspectives of mental health peer supporters of older adults and service users. There is considerable uncertainty about stakeholders’ expectations of peer supporters, especially in a non-Western context.^15,16^ Western views on peer supporter competencies may not be entirely applicable owing to unique aspects of Chinese culture, such as a disinclination to seek formal help for mental conditions because of perceived stigmatisation or guilt of burdening others.^17,18^ A clearer and accurate understanding of expected competencies is vital for the development of local peer support programmes.^14,19^

The main aims of the current study were to achieve consensus on the core competencies of mental health peer supporters of older adults and conceptualise a core competency framework for practical and research purposes. We used a bottom-up Delphi method to systematically acquire a grounded understanding of the perspectives of peer supporters, service users and helping professionals in Hong Kong to offer insight into the core competencies of peer supporters in a Chinese context with key comparisons with Western literature.

## Method

### Delphi method

The Delphi method is an established structural process for collecting and aggregating informed judgements from a panel of stakeholders on a specific topic and is especially suitable for emergent fields of inquiry.^20,21^ Conventionally, participants complete iterative ‘rounds’ of questions interspersed with controlled feedback on responses to assess or reach consensus among the panel.^22,23^ Stakeholder opinions are gathered anonymously to limit group influence and maximise objectivity.^24^ The Delphi method can utilise technological advances and cater to particular research objectives or settings,^25,26^ and it is particularly useful here given the limitation of current knowledge and consensus of peer support topics in non-Western countries.^26,27^ As our study was conducted during the COVID-19 pandemic, the method offered the particular advantages of time flexibility and not having to physically interact with participants.

**Fig. 1:**
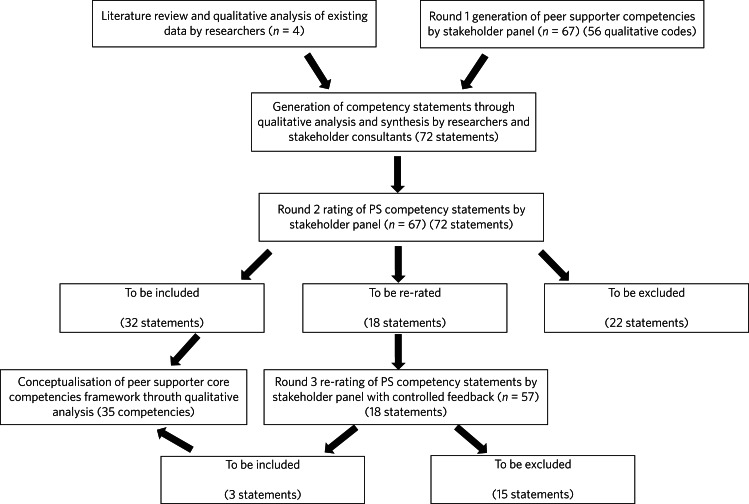
Delphi study steps and rounds.

We conducted a modified three-round bottom-up Delphi study (Fig. 1). The study adopted all conventional research ethics protocols and was approved by the Human Research Ethics Committee of the University of Hong Kong (reference number EA210166). Written informed consent was obtained from all participants. In round 1, participants generated important competencies of peer supporters for older adults with minimal guidance from the research team. We then developed a list of competency statements through qualitative analysis and synthesis of the collected responses, key Western literature and data from a local mental health peer support project. In Rounds Two and Three, participants rated the importance of each competency statement. Finally, we used quantitative criteria to determine a consensus regarding the importance of each competency statement and conceptualised a core competency framework based on themes identified from the included statements.

### Preparatory steps

Before involving participants, we conducted a brief review of key literature on peer supporter competencies and qualitative analysis of self-reported service experiences of 153 peer supporters from a territory-wide peer support project in Hong Kong.^4^ The project recruited and trained ‘young-old’ (i.e. aged 50 years and over) peer supporters with interests in mental health or personal history of mental illness to support older people at risk of or with subthreshold depression. Supplementary Table 1, available at https://dx.doi.org/10.1192/bjb.2023.45, provides details of our preparatory steps. We aimed to assess existing knowledge on the research topic and establish a foundation for cross-cultural comparison and integration of peer supporter competencies derived from Western research and this study.

### Participants

Participants were recruited from three stakeholder groups: peer supporters, service users and helping professionals (i.e. mental health social workers and peer support project officers) who could effectively communicate in Chinese or Cantonese and possessed any experience with peer support. The minimum age requirement for service users was 60 years. We aimed to recruit at least ten participants from each group to achieve response stability, as previous Delphi studies on mental health suggested.^28,29^ All participants were identified and recruited from the JC JoyAge project. No monetary compensation was given for participation.

### Round 1: generation of competency statements

Participants answered demographic questions and were asked to list at least five important competencies for mental health peer supporters for older adults, with the option to elaborate on the underlying reasons for their choices. Supplementary information was provided about mental health peer support for older adults and a broad definition of their competencies with generic examples. We used thematic analysis to categorise conceptually similar responses into unique competency themes. Following repeated comparison and integration with competency themes identified from Western literature and service experiences data in the preparatory steps, we compiled a comprehensive list of 72 competency statements. Wordings followed the original sources, with adaptations and examples to accommodate the local context and minimise ambiguity. The draft list was reviewed for descriptive appropriateness by two expert consultants and refined by our team before finalisation.

### Round 2: initial rating of competency statements

Participants rated the importance of the 72 competency statements on a seven-point Likert scale with an option to provide additional open-text comments. We adopted consensus criteria based on proportion scores and measures of central tendency to ensure robustness in evaluating the consensus levels on the importance of each competency statement.^13,30^ A statement achieved positive consensus when (a) ≥80% of participants rated it 6 or 7 and (b) the interquartile range (IQR) was ≤1, and it was included in the competency framework. A statement failed to achieve positive consensus when <70% of participants rated it 6 or 7, and it was therefore excluded from the framework. A statement met borderline consensus if 70–79% of participants rated it 6 or 7, and it was listed for re-rating in round 3.

### Round 3: re-rating of competency statements

Participants re-rated borderline statements using the same seven-point Likert scale. We provided the mode and range of round 2 scores for each statement. Statements that met the consensus criteria (i.e. ≥80% of participants scored it 6 or 7 and IQR ≤ 1) were included in the competency framework and the remaining statements were excluded.

### Conceptualisation of the peer supporter core competency framework

We qualitatively analysed competency statements achieving a positive consensus to identify overarching themes and subthemes, organised into a conceptual framework based on relative specificity for the peer supporter role as evidenced by the literature.^11,12^

### Data collection and analysis

For all rounds, we mainly utilised the questionnaire platform Qualtrics for data collection, conducting phone and in-person interviews with service users to accommodate their lower literacy levels.

Quantitative data analysis was conducted using IBM SPSS Statistics (Version 26) for Windows. Four researchers with psychology or social science training backgrounds (E.L.Y.W., J.H.-Y.Y., L.C.Y.S. and H.S.K.) conducted the qualitative analysis to identify themes and subthemes for the competencies in all three rounds, generating competency statements and conceptualising the core competency framework. At least two researchers worked on each qualitative step to reduce personal biases, and differences were resolved through group discussion and decision-making. T.Y.S.L. and T.L. oversaw the overall project.

## Results

### Participants’ characteristics

Around 200 stakeholders were invited to participate in the study: 67 participated in round 1, 60 in round 2 and 57 in round 3 – generating an acceptable final attrition rate of 14.9%.^31^ Table 1 provides details of participants’ demographic characteristics in round 1.

**Table 1:** Round 1 participants’ characteristics

Characteristic	Overall stakeholder panel *n* = 67 (100%)	Peer supporter *n* = 30 (44.8%)	Service user *n* = 14 (20.9%)	Helping professional *n* = 23 (34.3%)
Gender, *n* (%)				
Female	50 (74.6)	23 (76.7)	12 (85.7)	15 (65.2)
Male	17 (26 = 5.3)	7 (23.3)	2 (14.3)	8 (34.8)
Age, years: mean (s.d.)	56.4 (16.8)	62.4 (5.76)	74.5 (6.04)	35 (7.73)
Age group, *n* (%)				
20–49 years	19 (29.2)	0 (0)	0 (0)	19 (90.5)
50–59 years	10 (15.4)	8 (26.7)	0 (0)	2 (9.5)
60–69 years	21 (32.3)	18 (60)	3 (21.4)	0 (0)
≥70 years	15 (23.1)	4 (13.3)	11 (78.6)	0 (0)
Education level, *n* (%)				
No formal education	3 (4.5)	0 (0)	3 (21.4)	0 (0)
Primary school	4 (6.1)	4 (13.3)	4 (28.6)	0 (0)
Secondary school	25 (37.9)	13 (43.4)	7 (50)	1 (4.5)
Diploma or above	34 (51.5)	13 (43.4)	0 (0)	21 (95.5)
Experience related to peer support, years: mean (s.d.)	2.2 (2)	2.57 (2.09)	0.96 (0.71)	2.47 (2.28)
Lived experience of clinical depression, *n* (%)	12 (18.2)	6 (20)	6 (42.9)	0 (0)

### Results of round 1

Participants provided 404 individual answers for peer supporter competencies they perceived as important. Fifty-six competency-related codes were extracted following thematic analysis, encompassing peer support-specific expertise, capabilities of helping para-professionals, and general interpersonal or work skills and attitudes. Codes unique to the local context were mostly related to work ethics. Through iterative refinement and synthesis with local and Western competency themes identified in the preparatory steps, we generated 72 distinct competency statements for consensus rating in subsequent rounds. Regarding their conceptual origin, 7 (9.7%) statements were local, 40 (55.6%) Western and 25 (34.7%) local and Western.

### Results of round 2

Supplementary Table 2 details the consensus rating results of all competency statements in round 2. Thirty-two (44.4%) statements met the positive consensus criteria on the importance and were included in the framework. These statements mostly related to interpersonal or communication skills, work ethics and self-management and had either local or local and Western conceptual origins. Conversely, 22 (30.6%) statements failed to meet the positive consensus criteria and were excluded. The remaining 18 (25%) achieved borderline consensus and were re-rated in round 3.

Overall, the competency statement with the highest consensus on importance was ‘Possess a sense of responsibility for peer support work’, followed by ‘Possess listening skills’ and ‘Be able to abide by confidentiality principles’. The three competency statements securing the least consensus were ‘Be able to provide support to service users’ families and caregivers’, ‘Possess rich volunteering or other support provision experience’ and ‘Be able to bring recovery concepts into different fields as a leader’.

‘Sense of responsibility’ and ‘Listening skills’ achieved the highest consensus among peer supporters. ‘Possess care and love towards service users’ and ‘Be willing to accompany service users in the role of a peer’ achieved the highest consensus among service users. ‘Be able to appropriately care for service users’, ‘Possess a sincere attitude’ and ‘Be able to support service users in engaging in personally meaningful events’ achieved the highest consensus among helping professionals.

### Results of round 3

Supplementary Table 3 describes the consensus re-rating results of the 18 borderline competency statements in round 3. Three – ‘Be able to understand the different needs of service users’, ‘Be able to utilise physical and mental health knowledge to support service users’ and ‘Be able to maintain their proactiveness in peer support work’ – met the positive consensus criteria and were added to the framework. The remaining 15 failed to meet the required threshold and were excluded.

### Peer supporter core competency framework

**Fig. 2:**
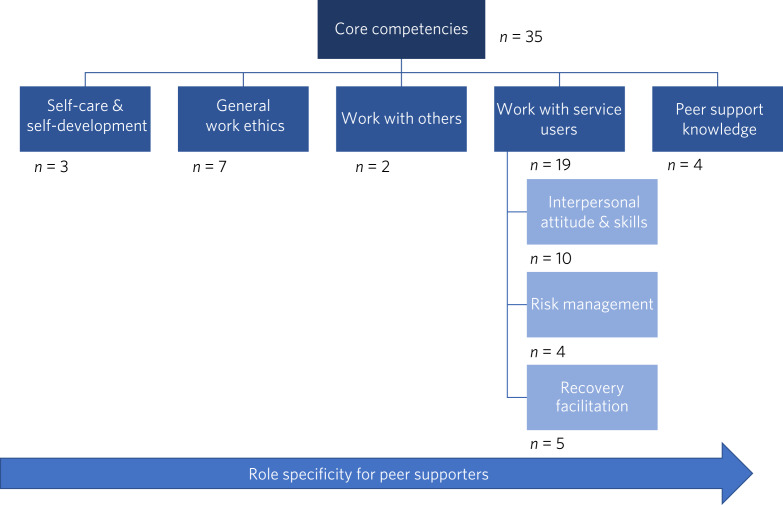
Peer supporter core competencies framework.

Figure 2 displays our core competency framework with five peer supporter competency categories arranged on a spectrum of role specificity, from general competencies required of any typical helping volunteer or personnel on the left to relatively specialised competencies on the right. Table 2 outlines the 35 competency statements incorporated within the framework.

**Table 2:** Core competence statements included after three Delphi rounds

Category and conceptual origins^a^
Self-care & self-development
Be able to manage their own emotions. [L + W] Be able to maintain their own physical and mental wellness. [L] Be able to utilise different methods to continually grow and enhance their work performance (e.g. consult colleagues or others about their opinions). [L + W]
General work ethics
Possess a sense of responsibility for peer support work (e.g. be trustworthy, inform others of decisions in a timely fashion). [L] Be able to abide by confidentiality principles. [L + W] Be willing to spend and arrange their time. [L] Be able to remain humble during peer support work. [L] Be passionate towards or feel interested in peer support work. [L + W] Be able to abide by the different rules and guidelines of service organisations or projects. [W] Be able to maintain their proactiveness in peer support work (e.g. actively contact target participants). [L + W]
Work with others
Be able to work together as a team (e.g. with peer supporters and non-peer staff). [L + W] Be able to grasp opportunities to review their peer support work with a team (e.g. share experiences and learn from each other). [W]
Work with service users
Subcategory: interpersonal attitude & skills
Possess listening skills (e.g. pay full attention to the needs of service users). [L + W] Possess care and love towards service users. [L + W] Know not to carelessly criticize or chide users. [L + W] Have empathy (e.g. be able to understand the experiences and feelings of service users by stepping into their shoes). [L + W] Be able to appropriately care for service users. [L + W] Possess a sincere attitude. [L + W] Be able to provide a sense of security to service users. [L + W] Be willing to accompany service users in the role of a peer. [L + W] Be able to accept and respect service users’ different situations and backgrounds; for example, their religion, culture or identity. [L + W] Possess communication skills (e.g. interact adequately with service users). [L + W]
Subcategory: risk management
Be able to monitor service users’ physical and mental circumstances. [L + W] Be aware of service users’ self-harm or suicidal risk. [W] Be resourceful (e.g. be able to deal with sudden events during service provision). [L] Be able to support service users in dealing with emergency situations (e.g. enable service users to feel safe; know how to adopt emergency contingency procedures). [W]
Subcategory: recovery facilitation
Be able to understand the different needs of service users. [L + W] Be able to utilise physical and mental health knowledge to support service users. [L + W] Be able to encourage and support a positive mindset and proactive behaviour among service users. [L + W] Be able to support service users in utilising their strengths and skills. [L + W] Be able to utilise positive mindsets to encourage service users. [L + W]
Peer support knowledge
Be able to grasp their role in peer support work. [W] Agree with peer support ideals, including supporting service users experiencing emotional distress as a companion. [L + W] Be familiar with the work and moral ethics of peer supporters. [W] Understand the need to acquire service users’ consent during peer support work (e.g. agree to join centre activities together). [W]

a. L, local; W, Western; L + W, local and Western.

## Discussion

This is the first Delphi study to determine consensus among peer supporters, service users and helping professionals on the competencies they perceive most important for mental health peer supporters of older adults in a Chinese context, resulting in the generation of 35 competency statements. We conceptualised themes from these statements into five categories of competency and organised them into a core competency framework based on peer supporter role specificity. The category incorporating the least role specificity, ‘Self-care and self-development’, includes competencies relating to peer supporters’ ability to manage their own physical or mental health and grow as a service provider. Next, the ‘General work ethics’ category sets out the universal attitudes and values peer supporters require regarding work responsibilities. The ‘Work with others’ category delineates competencies peer supporters need to work alongside team members effectively. Fourth, ‘Work with service users’ comprises three subcategories indicating the range of competencies required to support target individuals: (a) the ‘Interpersonal attitude and skills’ subcategory entails communication and relational competencies needed when working with service users; (b) the ‘Risk management’ subcategory outlines competencies for assisting service users before or during emergencies; (c) the ‘Recovery facilitation’ subcategory describes competencies that help drive the unique personal recovery process and service users’ goals. Fifth, the ‘Peer support knowledge’ category comprises the greatest role specificity, encapsulating peer supporters’ holistic understanding of their line of service.

### Comparison with the Western literature

Many competencies viewed by participants as important mirrored those in Western literature, although particular competencies were widely agreed as important only in the local context. These included ‘Possess a sense of responsibility for peer support work’, ‘Be willing to spend and arrange their time’, ‘Be able to remain humble during peer support work’ and ‘Be resourceful’. This suggests that peer supporters in Hong Kong may have embodied the role of generic support companions possessing a respectful attitude and being responsible and devoted. Indeed, statistical analysis revealed that ‘General work ethics’ and the subcategory ‘Interpersonal attitude and skills’ largely secured high scores. For local stakeholders, peer supporters most prominently served as trusted, objective and professional ‘befrienders’. This notion corresponds with previous research suggesting that Chinese service users felt more at ease sharing with or soliciting the help of peer supporters rather than family members or other helping professionals.^18^ Interactions were deemed safer and more equal, leaving service users less burdened or feeling guilty about their mental health problems.

Numerous competencies related to peer support ideology and values from Western sources were excluded, suggesting that a thorough understanding and recognition of the concepts and principles underpinning peer support are currently deemed less important in Hong Kong. Local training for peer supporters has emphasised the attitudes and relational skills required when working with service users and helping build social connections and links to resources to enhance mental wellness.^31^ In comparison, comprehensive job training and descriptions for peer supporters were more abundant in Western contexts.^32^ Contemporary peer support modules have been designed to support guidelines under wider mental health recovery policy directives.^33,34^ To cater for cultural differences, local peer supporter training could be refined to focus more on non-specific interpersonal skills and work ethics to better address the uniqueness of the Hong Kong population's needs. Another cultural explanation is that the stigma of mental illness remains pervasive in Chinese society, and stakeholders are unfamiliar with recovery concepts and practices.^35,36^ Therefore, competencies entrenched in Western peer support ideology and values may not be as instrumental in helping Hong Kong service users.

There was no consensus on the importance of competencies related to role modelling, hypothesised as a change mechanism for peer support services.^37^ This may be because Chinese older adults are less inclined than their younger counterparts to seek a role model for or reference others’ recovery journeys, whether this is due to inherently richer life experiences, concerns to save face or stigma regarding help-seeking.^38,39^ In addition, our framework does not include advocacy or leadership competencies considered advanced capacities by Western counterparts. A plausible reason for this is the nascent development of mental health peer support practice in Hong Kong, leading stakeholders not yet to view promoting services, and their recovery-oriented mission or rights, as essential tasks for peer supporters.^40^

### Strengths and limitations

A major strength of this study is the broad representativeness of the sample and inclusion of three stakeholder groups, which helped counterbalance the researchers’ potential reflexivity and afforded high validity regarding the competencies perceived as core locally. Our first-hand experience reflected the importance of involving older Chinese adults in creating a list of competency statements. Their understanding, experiences and areas of interest often differ from those of academics and helping professionals, especially those from Western contexts. Another strength was the low attrition rate, suggesting that most participants were invested in the generation, rating and re-rating of competencies. Over half of the competency statements failed to achieve overall consensus at the end of the study, indicating that participants were willing to disclose individual opinions and not prepared simply to accept the stakeholder panel's perspectives.

A limitation was the potential confirmatory biases held by stakeholders who chose to join this no-rewards study. Although no observed differences in characteristics between participants and non-participant invitees were evident, the former may have a greater underlying commitment to or better experiences of peer support. Moreover, most round 1 responses lacked elaboration, which did not allow for extensive analysis of stakeholders’ viewpoints. The results might not be readily transferable to other regions or peer support fields, given that all participants were recruited via a Hong Kong project on older adult mental wellness. Nonetheless, our modified Delphi design incorporating flexible modes of data collection should be easy to replicate and adapt to examine consensus on peer supporter competencies in different contexts.

### Implications

Our competency framework can promote general advancement in the field, especially in a Chinese context, by serving as a guide for stakeholders to recalibrate expectations and practices for peer supporter recruitment, training and supervision. Role confusion and conflict in peer support can be minimised as peer supporters, service users and helping professionals can acquire a solid grasp of an ideal peer supporter's core competencies and, therefore, their expected service nature and goals. Although no distinction is made between the relative importance or proficiency level required of competency categories, stakeholders can adapt their service and training mindsets, goals and decision-making based on personal backgrounds or specific contexts. The framework does not seek to exclude or limit individuals from becoming peer supporters and encourages their development of core competencies and self-monitoring of service provision.

The results should not be treated as a recommendation that local peer supporters retreat to the basic role of a generic support worker, nor a dismissal of competencies seen as important in the West but not included within the framework. Owing to our adoption of strict consensus criteria, many excluded competencies were still regarded as important by a simple majority of stakeholders (i.e. rated as very important or extremely important by >50% of participants). The potential next steps for research will be in-depth qualitative investigation of peer supporter competencies (e.g. interviews and focus groups with various stakeholder groups) to ascertain the rationales behind differences in consensus. This can help fine-tune the framework and develop peer support practice.

## About the authors

**Edwin Lok Yan Wong** is a senior research assistant in the Department of Social Work and Social Administration, The University of Hong Kong, Hong Kong. **Jessie Ho-Yin Yau** is a PhD candidate in the Department of Social Work and Social Administration, The University of Hong Kong, Hong Kong. **Lesley Cai Yin Sze** is a senior research assistant in the Department of Social Work and Social Administration, The University of Hong Kong, Hong Kong. **Hotinpo Sky Kanagawa** is a research assistant in the Department of Social Work and Social Administration, The University of Hong Kong, Hong Kong. **Dara Kiu Yi Leung** is a Postdoctoral Fellow in the Department of Social Work and Social Administration, The University of Hong Kong, Hong Kong. **Tianyin Liu** is a research assistant professor in the Department of Social Work and Social Administration, The University of Hong Kong, Hong Kong. **Gloria Hoi Yan Wong** is an associate professor in the Department of Social Work and Social Administration, The University of Hong Kong, Hong Kong. **Terry Yat Sang Lum** is a professor in the Department of Social Work and Social Administration, The University of Hong Kong, Hong Kong, and at the Sau Po Centre on Ageing, The University of Hong Kong, Hong Kong.

## Supporting information

Wong et al. supplementary material Wong et al. supplementary material

## Data Availability

The data that support the findings of this study are available from the corresponding author, T.Y.S.L., on reasonable request.
