# Supplementary material for: A core competency framework for mental health peer supporters of older adults in a Chinese community: cross-culturally informed Delphi study
Source: BJPsych Bull. 2024 Apr;48(2):92–9. doi: 10.1192/bjb.2023.45 (PMC10985733; doi:10.1192/bjb.2023.45)
Supplement: Wong et al. supplementary material [file S2056469423000451sup001.docx]

**Supplementary Materials**

**Supplementary Table 1. Literature/Resources Examined during Preparatory Steps**

| **Key Western literature reviewed** | Burke E M, Pyle M, Machin K, Morrison A P. Providing mental health peer support 1: A Delphi study to develop consensus on the essential components, costs, benefits, barriers and facilitators. Int J Soc Psychiatry 2018; **64**(8): 799-812.  Campos F, Sousa A, Rodrigues V, Marques A, Queirós C, Dores A. Practical guidelines for peer support programmes for mental health problems. Rev Psiquiatr (English Edition) 2016; **9**(2): 97-110.  Lakeman R.  Mental health recovery competencies for mental health workers: a Delphi study. J Ment Health 2010; **19**(1): 62-74.  Moran G S. A recovery-oriented peer provider (ROPP) work-role model and prototype measure. Am J Psychiatr Rehabil 2017; **20**(4): 346-368.  National Association of Peer Supporters. National Practice Guidelines for Peer Specialists and Supervisors. Washington, DC: National Association of Peer Supporters, 2019.  National Health Service. The competence framework for mental health peer support workers, 2020.  Retrieved from [https://www.hee.nhs.uk/sites/default/files/documents/The%20Competence%20Framework%20for%20MH%20PSWs%20-%20Part%202%20-%20Full%20listing%20of%20the%20competences.pdf](about:blank)  Repper  J,  Aldridge  B,  Gilfoyle  S,  Gillard  S,  Perkins  R,  Rennison  J.  Peer  support  workers: a practical  guide to implementation (Briefing  Paper  7).  Implementing recovery through organisational change, 2013*.* Retrieved   from [https://imroc.org/resources/5-peer-sup-port-workers-theory-practice/](about:blank)  Substance Abuse and Mental Health Service Administration.  Core competences for peer workers in behavioral health services, 2015.  Retrieved from [https://www.opioidlibrary.org/wp-content/uploads/2021/01/core-competencies_508_12_13_18.pdf](about:blank) |
| --- | --- |
| **Details of existing project data on mental health peer supporters for older adults** | Sample: 153 older peer supporters (mostly aged >50 years) from a Hong Kong territory-wide project to tackle older adult depression and raise awareness regarding their mental health  Form of data: Qualitative responses in Chinese to questions regarding peer supporters’ service experiences (e.g., ‘What difficulties or challenges do you face when interacting with older adults [service users]?’; ‘During you first visit, what did you do to help yourself understand the older adult [service user] and establish rapport?’)  Examples of peer supporter competences identified: empathy and acceptance; listening/communication skills; observation skills; ability to encourage; perseverance; self-care |

**Supplementary Table 2. Results of Delphi Round 2**

| **Variables** | **Origin** | **Overall stakeholder panel** | **Participants by role** | | | **Level of consensus on importance** |
| --- | --- | --- | --- | --- | --- | --- |
|  |  |  | **Peer supporter** | **Service user** | **Helping professional** |  |
| **Population, n (%)** | / | 60 (100) | 27 (45) | 13 (21.7) | 20 (33.3) | / |
| **Competence statements, proportion scores^a^ (IQR** ^b^**)**  "An ideal mental health peer supporter for older adults should…" |  |  |  |  |  |  |
| Possess a sense of responsibility for peer support work (e.g., be trustworthy, timely inform others of decisions). | L ^c^ | 95 (1) | 100 | 85 | 95 | Achieved |
| Possess listening skills (e.g., pay full attention to service users’ needs). | L + W | 93 (1) | 100 | 77 | 95 | Achieved |
| Be able to abide by confidentiality principles. | L + W | 93 (1) | 96 | 92 | 90 | Achieved |
| Possess care and love towards service users. | L + W | 92 (1) | 93 | 92 | 90 | Achieved |
| Be able to manage their own emotions. | L + W | 92 (1) | 93 | 83 | 95 | Achieved |
| Know not to carelessly criticize or chide users. | L + W | 92 (1) | 96 | 83 | 90 | Achieved |
| Have empathy (e.g., be able to understand the experiences and feelings of service users by stepping into their shoes). | L + W | 90 (1) | 93 | 77 | 95 | Achieved |
| Possess a sincere attitude. | L + W | 90 (1) | 96 | 62 | 100 | Achieved |
| Be able to appropriately care for service users. | L + W | 90 (1) | 85 | 83 | 100 | Achieved |
| Be able to provide a sense of security to service users. | L + W | 88 (1) | 93 | 69 | 95 | Achieved |
| Be able to maintain their own physical and mental wellness. | L | 88 (1) | 96 | 75 | 85 | Achieved |
| Be able to work together as a team (e.g., with peer supporters and non-peer staff). | L + W | 88 (1) | 85 | 83 | 95 | Achieved |
| Be able to grasp their role in peer support work. | W | 86 (1) | 81 | 83 | 95 | Achieved |
| Be willing to accompany service users in the role of a peer. | L + W | 86 (1) | 85 | 92 | 85 | Achieved |
| Be familiar with the work and moral ethics of peer supporters. | W | 85 (1) | 93 | 69 | 85 | Achieved |
| Be able to monitor service users’ physical and mental circumstances. | L + W | 85 (1) | 93 | 77 | 80 | Achieved |
| Agree with peer support ideals, including supporting service users experiencing emotional distress as a companion. | L + W | 85 (1) | 85 | 75 | 90 | Achieved |
| Understand the need to acquire service users' consent during peer support work (e.g., agree to join centre activities together). | W | 85 (1) | 89 | 75 | 85 | Achieved |
| Be willing to spend and arrange their time. | L | 83 (1) | 89 | 69 | 85 | Achieved |
| Be aware of service users’ self-harm or suicidal risk. | W | 83 (1) | 93 | 69 | 80 | Achieved |
| Be able to remain humble during peer support work. | L | 83 (1) | 81 | 75 | 90 | Achieved |
| Be passionate about and feel interested in peer support work. | L + W | 83 (1) | 85 | 75 | 85 | Achieved |
| Be able to utilise different methods to continually grow and enhance their work performance (e.g., consult colleagues or others about their opinions). | L + W | 83 (1) | 81 | 83 | 85 | Achieved |
| Be able to accept and respect service users' different situations and backgrounds; for example, their religion, culture or identity. | L + W | 83 (1) | 93 | 69 | 80 | Achieved |
| Be able to abide by the different rules and guidelines of service organizations or projects. | W | 82 (1) | 89 | 62 | 85 | Achieved |
| Be resourceful (e.g., be able to deal with sudden events during service provision). | L | 81 (0) | 78 | 83 | 85 | Achieved |
| Possess communication skills (e.g., interact adequately with service users). | L + W | 80 (1) | 78 | 69 | 90 | Achieved |
| Be able to utilise positive mindsets to encourage service users. | L + W | 80 (1) | 93 | 62 | 75 | Achieved |
| Be able to support service users in dealing with emergency situations (e.g., enable service users to feel safe, know how to adopt emergency contingency procedures). | W | 80 (1) | 85 | 67 | 80 | Achieved |
| Be able to grasp opportunities to review their peer support work with a team (e.g., share experiences and learn from each other). | W | 80 (0) | 85 | 58 | 85 | Achieved |
| Be able to encourage and support the positive mindset and proactive behaviour of service users. | L + W | 80 (1) | 81 | 83 | 75 | Achieved |
| Be able to support service users in utilizing their strengths and skills. | L + W | 80 (1) | 81 | 75 | 80 | Achieved |
| Be able to maintain their proactiveness in peer support work (e.g., actively contact target participants). | L + W | 78 (1) | 78 | 69 | 85 | Borderline |
| Be able to support service users in engaging in personally meaningful events. | W | 78 (1) | 67 | 67 | 100 | Borderline |
| Be willing to continually learn to maintain the quality of their peer support work. | L + W | 78 (1) | 93 | 54 | 75 | Borderline |
| Be persistent towards peer support work. | L | 77 (1) | 81 | 85 | 65 | Borderline |
| Acknowledge that recovery-orientation and person-centredness are the main ideals of peer support. | W | 77 (1) | 89 | 62 | 70 | Borderline |
| Be able to understand the different needs of service users. | L + W | 77 (1) | 81 | 54 | 85 | Borderline |
| Be able to assist service users in developing coping and problem-solving skills. | W | 75 (1) | 70 | 77 | 80 | Borderline |
| Be aware when service users' safety is threatened by others (e.g., being abused). | W | 75 (2) | 85 | 58 | 70 | Borderline |
| Be able to discuss and explore with service users the support services they need. | W | 75 (2) | 74 | 75 | 75 | Borderline |
| Be able to make use of social resources to support target participants. | L + W | 73 (2) | 78 | 77 | 60 | Borderline |
| Possess good memory (e.g., be able to remember the personal sharing of service users). | L | 73 (2) | 81 | 69 | 65 | Borderline |
| Be able to strengthen service users' ties with the community. | L + W | 73 (2) | 74 | 75 | 70 | Borderline |
| Be able to support service users during the transition to other services or care models. | W | 72 (2) | 78 | 46 | 80 | Borderline |
| Be able to develop their peer support work (e.g., participate in training). | W | 72 (2) | 85 | 69 | 55 | Borderline |
| Be willing to continually acquire knowledge and skills. | L + W | 71 (2) | 78 | 58 | 70 | Borderline |
| Be able to provide personalized peer support according to the characteristics of target participants (e.g., background and personality). | L + W | 70 (2) | 78 | 54 | 70 | Borderline |
| Be able to instill hope in service users regarding the progress of their recovery. | L + W | 70 (2) | 70 | 77 | 65 | Borderline |
| Be able to utilise physical and mental health knowledge to support service users. | L + W | 70 (1) | 85 | 77 | 45 | Borderline |
| Be able to utilise the unique aspects of being a peer when carrying out peer support work (e.g., share similar experiences with service users as someone who has been there). | L + W | 69 (1) | 59 | 75 | 80 | No consensus |
| Be able to reflect on their peer support work (e.g., challenges and feelings during service provision). | W | 69 (1) | 78 | 58 | 65 | No consensus |
| Be able to support service users who have experienced psychological or other trauma before (e.g., consider the physical and psychological impact of the trauma and post-traumatic responses ). | L + W | 68 (2) | 78 | 62 | 60 | No consensus |
| Be able to work collaboratively with service users to identify possible recovery methods (e.g., set up and accomplish personal targets related to family, work and health). | L + W | 68 (2) | 85 | 54 | 55 | No consensus |
| Be able to facilitate the rights of service users (e.g., support service users to express their personal opinions). | W | 68 (2) | 74 | 83 | 50 | No consensus |
| Be able to pull away from the role of a peer supporter. | L + W | 67 (2) | 85 | 31 | 65 | No consensus |
| Be able to model self-care and recovery strategies (e.g., demonstrate the utility of different coping methods to service users by utilizing personal experiences). | L + W | 66 (2) | 59 | 75 | 70 | No consensus |
| Be able to reflect on their strengths and weaknesses. | L + W | 65 (2) | 74 | 31 | 75 | No consensus |
| Be able to enhance service users’ autonomy and motivation (e.g., support different decisions made by service users for their own lives). | W | 63 (2) | 63 | 54 | 70 | No consensus |
| Be able to facilitate service users’ receipt of appropriate care services and support. | W | 59 (2) | 67 | 75 | 40 | No consensus |
| Be able to share personal experiences and recovery stories with service users. | L + W | 58 (2) | 63 | 62 | 50 | No consensus |
| Be able to utilise basic psychological skills to support service users' recovery (e.g., counselling skills). | W | 58 (1) | 59 | 75 | 45 | No consensus |
| Be able to promote the values of peer support (e.g., advocate person-centred and recovery-oriented approaches). | W | 57 (2) | 67 | 62 | 40 | No consensus |
| Be able to share their own knowledge with service users. | W | 56 (1) | 59 | 50 | 55 | No consensus |
| Be able to handle written and paper work (e.g., complete written records in accordance with standards of the service project). | L + W | 55 (1) | 52 | 62 | 55 | No consensus |
| Be able to develop and set customized care or recovery plans with service users. | W | 55 (1) | 52 | 46 | 65 | No consensus |
| Be able to help non-peer support workers understand the uniqueness of individual recovery. | W | 53 (1) | 63 | 54 | 40 | No consensus |
| Be able to offer practical daily life assistance to service users (e.g., accompany them on hospital appointments). | L + W | 52 (1) | 44 | 69 | 50 | No consensus |
| Be able to support service users' use of information technology. | L + W | 47 (2) | 52 | 50 | 40 | No consensus |
| Be able to bring recovery concepts into different fields as a leader. | W | 45 (2) | 52 | 62 | 25 | No consensus |
| Possess rich volunteering or other support provision experience. | L + W | 40 (2) | 44 | 69 | 15 | No consensus |
| Be able to support service users' families and caregivers. | W | 32 (2) | 30 | 46 | 25 | No consensus |

a Proportion scores = % of participants who answered 6 or 7; displayed in descending % of rating by all participants

b IQR = Interquartile range

c L = local, W = Western, L + W = local + Western

**Supplementary Table 3. Results of Delphi Round 3**

| **Variables** | **Origin** | **Overall stakeholder panel** | **Participants by role** | | | **Level of consensus on importance** |
| --- | --- | --- | --- | --- | --- | --- |
|  |  |  | **Peer supporter** | **Service user** | **Helping professional** |  |
| **Population, n (%)** | / | 57 (100) | 26 (45.6) | 12 (21.1) | 19 (33.3) | / |
| **Competence statements, proportion scores^a^ (IQR** ^b^**)**  "An ideal mental health peer supporter for older adults should…" |  |  |  |  |  |  |
| Be able to understand the different needs of service users. | L + W ^c^ | 88 (1) | 89 (1) | 92 | 84 | Achieved |
| Be able to utilise physical and mental health knowledge to support service users. | L + W | 83 (0) | 96 (0) | 83 | 63 | Achieved |
| Be able to maintain their proactiveness in peer support work (e.g., actively contact target participants). | L + W | 81 (1) | 81 | 92 | 74 | Achieved |
| Be willing to continually acquire knowledge and skills. | L + W | 79 (1) | 92 | 67 | 68 | No consensus |
| Be able to support service users during the transition to other services or care models. | W | 77 (0) | 81 | 75 | 74 | No consensus |
| Be willing to continually learn to maintain the quality of their peer support work. | L + W | 77 (1) | 92 | 75 | 58 | No consensus |
| Acknowledge that recovery-orientation and person-centredness are the main ideals of peer support. | W | 77 (1) | 85 | 83 | 63 | No consensus |
| Be able to instill hope in service regarding the progress of their recovery. | L + W | 77 (1) | 73 | 92 | 74 | No consensus |
| Be able to support service users in engaging in personally meaningful events. | W | 77 (1) | 77 | 75 | 79 | No consensus |
| Be aware when service users' safety is threatened by others (e.g., being abused). | W | 77 (1) | 81 | 83 | 68 | No consensus |
| Be able to discuss and explore with service users the support services they need. | W | 77 (0) | 85 | 67 | 74 | No consensus |
| Be able to strengthen service users' ties with the community. | L + W | 75 (1) | 73 | 75 | 79 | No consensus |
| Be able to make use of social resources to support target participants. | L + W | 75 (1) | 81 | 77 | 68 | No consensus |
| Possess good memory (e.g., be able to remember the personal sharing of service users). | L | 75 (1) | 69 | 83 | 68 | No consensus |
| Be persistent towards peer support work. | L | 72 (2) | 81 | 67 | 63 | No consensus |
| Be able to develop their peer support work (e.g., participate in training). | W | 72 (2) | 85 | 67 | 58 | No consensus |
| Be able to provide personalized peer support according to the characteristics of target participants (e.g., background and personality). | L + W | 70 (2) | 77 | 58 | 68 | No consensus |
| Be able to assist service users in developing coping and problem-solving skills. | W | 65 (1) | 69 | 83 | 47 | No consensus |

a Proportion scores = % of participants who answered 6 or 7; displayed in descending % of rating by all participants

b IQR = Interquartile range

c L = local, W = Western, L + W = local + Western
